# The HALP score is a novel prognostic biomarker in multiple myeloma: A retrospective cohort study of 450 patients

**DOI:** 10.1097/MD.0000000000050093

**Published:** 2026-08-07

**Authors:** Gulsum Akgun Cagliyan, Ilhami Berber, Eda Nilufer Coskun, Suleyman Arslan, Huriye Karagul, Seher Yilmaz, Emin Kaya, Ismail Can Kendir, Basak Unver Koluman, Mehmet Ali Erkurt, Sibel Hacioglu, Aybuke Nur Ekici, Mehmet Nuri Baser, Irfan Kuku

**Affiliations:** a Department of Hematology, Pamukkale University Faculty of Medicine, Denizli, Turkey; b Department of Hematology, Inonu University, Turgut Ozal Medical Center, Malatya, Turkey; c Department of Internal Medicine, Pamukkale University Faculty of Medicine, Denizli, Turkey; d Department of Internal Medicine, Inonu University, Turgut Ozal Medical Center, Malatya, Turkey; e Department of Oncology, Faculty of Medicine, Aydin Adnan Menderes University, Aydin, Turkey.

**Keywords:** HALP score, multiple myeloma, prognosis, survival

## Abstract

This study aimed to evaluate the prognostic significance of hemoglobin, albumin, lymphocyte, and platelet (HALP) levels at diagnosis in patients with multiple myeloma (MM). We retrospectively analyzed 450 patients diagnosed with MM between 2008 and 2023. The HALP score was calculated using pretreatment laboratory values of hemoglobin, albumin, lymphocytes, and platelets. Patients were categorized into the high and low HALP groups. Survival outcomes were analyzed using the Kaplan–Meier and Cox regression methods. Patients with HALP ≤ 27.68 had a significantly lower overall survival (OS) (median 50 vs 79 months, *P* < .001). Patients with low HALP scores had significantly shorter median OS and progression-free survival than those with high HALP scores (*P* < .001). In the multivariate analysis, HALP ≤ 27.68 was independently associated with increased mortality risk (hazard ratio: 1.74; 95% confidence interavl: 1.32–2.29). The HALP score remained an independent predictor of OS. The HALP score is a useful, accessible, and cost-effective prognostic marker for MM, and may aid in risk stratification.

## 1. Introduction

Multiple myeloma (MM) is a disease arising from monoclonal proliferation of plasma cells within the bone marrow. MM accounts for approximately 1% of all cancers and 17% of all hematologic malignancies. It is the 2nd most common hematologic malignancy after lymphomas. It is characterized by osteolytic bone lesions, renal insufficiency, normochromic normocytic anemia, and hypercalcemia.^[1]^

Despite modern treatment modalities, MM remains an incurable disease. Treatment strategies are tailored according to the patient’s age, comorbidities, and performance status. The international staging system (ISS) is based on serum β2-microglobulin and albumin levels, while the revised ISS (R-ISS) further incorporates lactate dehydrogenase (LDH) levels and high-risk cytogenetic abnormalities to improve risk stratification in MM.^[2]^ Recent studies have shown that inflammatory indices, such as the neutrophil-to-lymphocyte ratio (NLR), lymphocyte-to-monocyte ratio (LMR), and platelet-to-lymphocyte ratio (PLR), are associated with the prognosis of hematologic malignancies, including Hodgkin and non-Hodgkin lymphoma, as well as MM.^[3,4]^

The hemoglobin, albumin, lymphocyte, and platelet (HALP) score is an immune-nutritional biomarker calculated using hemoglobin, albumin, lymphocyte, and platelet counts and was originally described in patients with gastric carcinoma.^[5]^ Platelet and lymphocyte counts in the HALP score provide information about immune status, whereas albumin and hemoglobin reflect nutritional status.^[5]^ The prognostic value of the HALP score has been demonstrated in various solid tumors, including gastric, colorectal, endometrial, pancreatic, prostate, small-cell lung, and biliary tract cancers.^[6]^ Recent studies have further confirmed its utility; for example, Li Q et al reported the prognostic and clinicopathological significance of HALP in non-small cell lung cancer, while Li J et al conducted an analysis highlighting HALP’s prognostic relevance across multiple solid tumors.^[7,8]^ However, the prognostic impact of the HALP score may vary across different cancer types; while lower HALP scores are generally associated with poorer outcomes in solid tumors, findings in hematological malignancies appear to be more heterogeneous.

In hematologic cancers, studies have evaluated HALP’s prognostic relevance in diffuse large B-cell lymphoma, Hodgkin lymphoma, and myelodysplastic syndromes.^[9–11]^ Additionally, Li N et al demonstrated the value of score in low-risk and early-stage extranodal natural killer/T cell lymphoma, emphasizing its applicability in hematologic malignancies.^[12]^ Given these findings, the HALP score was selected for investigation in this study due to its potential prognostic significance in malignancies, although its role in MM remains largely unexplored. Therefore, we aimed to evaluate the prognostic significance of the HALP score at diagnosis in patients with MM and assess its association with overall survival (OS) and progression-free survival (PFS). However, the interpretation of the HALP score in MM may be influenced by disease-specific factors such as bone marrow infiltration, immune dysfunction, and anemia related to plasma cell burden, highlighting the need for disease-oriented evaluation of this biomarker.

## 2. Materials and methods

This retrospective study was conducted between 2008 and 2023 at the Department of Hematology in Denizli, Pamukkale University Faculty of Medicine. Two different centers were included in the study. In total, 450 adult patients with newly diagnosed MM were included. Demographic data (age and sex), disease characteristics (stage and MM subtype), laboratory values, treatment regimens, and survival outcomes were extracted from the medical records. Baseline assessments performed prior to therapy initiation included a complete blood count, biochemistry panel (LDH, renal function tests, albumin, total protein, calcium, and β2-microglobulin), erythrocyte sedimentation rate, C-reactive protein, immunoglobulin quantification, serum free light chains, and serum/urine protein electrophoresis with immunofixation. Induction regimens included bortezomib/cyclophosphamide/dexamethasone, bortezomib/lenalidomide/dexamethasone, or daratumumab-based protocols. Serum biochemical parameters were analyzed using the same automated biochemistry analyzer and identical assay methods at both participating institutions, in accordance with the manufacturers’ instructions. Regular calibration and internal quality control procedures were performed throughout the study period to ensure analytical accuracy and reproducibility.

The HALP score was calculated using hemoglobin (g/L), albumin (g/L), absolute lymphocyte count (×10^9^/L), and platelet count (×10^9^/L). The original formula described by Chen et al as follows: hemoglobin (g/L) × albumin (g/L) × lymphocyte count (/L) divided by platelet count (/L).^[13]^ The HALP score was determined based on laboratory data obtained within 15 days prior to treatment.

### 2.1. Statistical analysis

All statistical analyses were performed using the IBM SPSS Statistics for Windows (version 25.0; IBM Corp.). Categorical variables are presented as counts and percentages, whereas continuous variables are expressed as mean ± standard deviation or median (min–max). Data normality was assessed using the Kolmogorov–Smirnov test (*P* > .05); accordingly, comparisons between HALP groups for continuous variables were conducted using independent *t* tests. Pearson chi-squared test was used to compare categorical variables. Receiver operating characteristic (ROC) curve analysis was performed to assess the predictive value of HALP score for mortality. The optimal HALP cutoff value was determined using ROC curve analysis based on Youden index to identify the threshold with maximum combined sensitivity and specificity. Furthermore, multivariate analyses were reviewed by an independent biostatistician to ensure the methodological accuracy. The Kaplan–Meier method was used to compare OS and PFS. Finally, multivariate Cox regression analyses were performed to evaluate the effects of various clinical variables on the mortality and progression risk. Statistical significance was set at *P* < .05.

## 3. Results

In total, 450 patients diagnosed with MM were included in this study. The cohort comprised 44.9% female and 55.1% male patients. According to the R-ISS staging, 16.9% of the patients were stage I, 24.4% were stage II, and 57.7% were stage III, based on albumin, β2-microglobulin, and cytogenetic results. The most common MM subtype was IGG-kappa (50.8%, n = 228). Autologous stem cell transplantation (ASCT) was performed in 53.1% of patients; the mean age among transplant recipients was 61.73 ± 9.77 years (median 63.0, range 40–86). The mean follow-up period was 35.55 ± 31.85 months. The sociodemographic and clinical characteristics of the patients are summarized in Table 1. 3.1. Subsection

**Table 1:** Sociodemographic characteristics and clinical data of the patients.

Variables	Total N = 450	HALP		*P*
		>27.68 N = 227	≤27.68 N = 223	
Age				
Mean ± SD	65.07 ± 10.69	63.49 ± 10.73	66.68 ± 10.43	.002†
Median (min–max)	65.0 (40–89)			
≤65	231 (51.3)	130 (57.3)	101 (45.3)	.011*
>65	219 (48.7)	97 (42.7)	122 (54.7)	
Sex				
Male	248 (55.1)	131 (57.7)	117 (52.5)	.264*
Female	202 (44.9)	96 (42.3)	106 (47.5)	
Albumin (g/dL)	3.40 ± 0.79	3.69 ± 0.74	3.12 ± 0.75	<.001†
Leukocytes (K/uL)	6.20 ± 3.13	6.44 ± 2.71	5.97 ± 3.5	.114†
Hemoglobin (g/dL)	10.71 ± 4.33	11.38 ± 5.73	10.04 ± 1.89	.001†
Platelets (K/µL)	193.23 ± 110.61	174.01 ± 94.64	212.8 ± 121.93	<.001†
Sedimentation (mm/h)	63.00 ± 42.60	59.37 ± 42.39	66.75 ± 42.6	.067†
Renal dysfunction				
No	234 (52.0)	132 (58.1)	102 (45.7)	.008*
Yes	216 (48.0)	95 (41.9)	121 (54.3)	
B2 microglobulin (mg/L)	7.26 ± 5.15	6.86 ± 5.1	7.72 ± 5.2	.161†
IGG	1257.15 ± 2006.92	1260.76 ± 2108.08	1253.51 ± 1903.66	.970†
IGA	431.54 ± 2314.40	474.27 ± 2969.67	388.05 ± 1360.82	.694†
IGM	30.23 ± 98.78	32.75 ± 75.85	27.68 ± 117.68	.588†
R-ISS stage				
Stage I	76 (16.9)	46 (20.3)	30 (13.5)	.153*
Stage II	110 (24.4)	54 (23.8)	56 (25.1)	
Stage III	264 (57.7)	127 (55.9)	137 (61.4)	
ASCT				
Not performed	211 (46.9)	87 (38.3)	124 (55.6)	.001*
Performed once	226 (50.2)	133 (58.6)	93 (41.7)	
Performed twice	13 (2.9)	7 (3.1)	6 (2.7)	
Progression				
No	135 (30.0)	57 (25.1)	78 (35)	.022*
Yes	315 (70.0)	170 (74.9)	145 (65)	
Mortality status				
Survivor	240 (53.3)	138 (60.8)	102 (45.7)	.001*
Deceased	210 (46.7)	89 (39.2)	121 (54.3)	
Follow-up period (mo)				
Mean ± SD	35.55 ± 31.85	46.24 ± 36.95	35.77 ± 34.82	.002†
Median (min-max)	27.8 (0.5–235.7)			

ASCT = autologous stem cell transplantation, HALP = hemoglobin, albumin, lymphocyte, and platelet, IGA = immunoglobulin A, IGG = immunoglobulin G, IGM = immunoglobulin M, R-ISS** = **revised international staging system, SD = standard deviation.

*P* < .05 statistically significant.

* Pearson chi square test.

† Independent samples *t* test.

**Figure 1.**
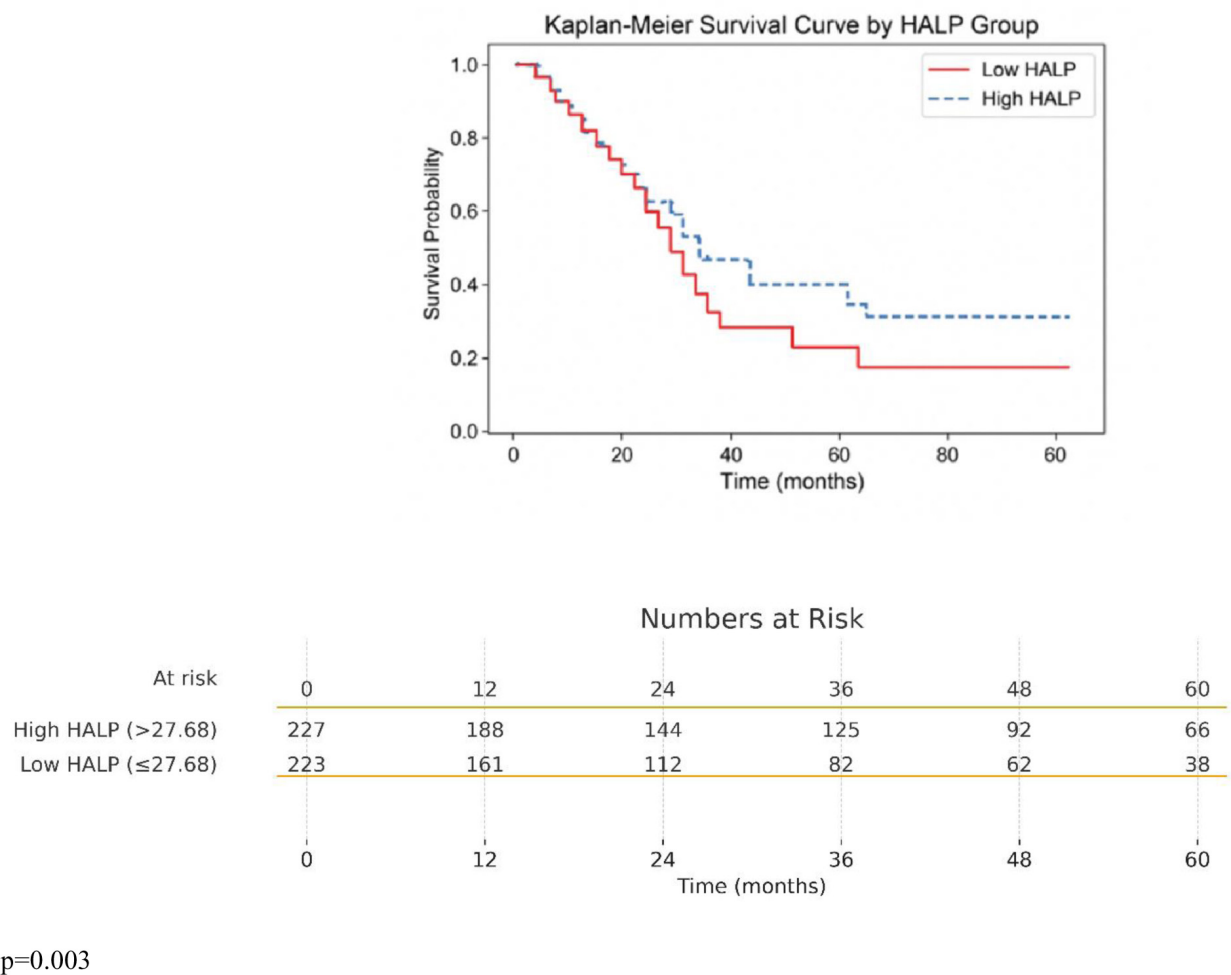
HALP score and survival (low or high). Kaplan–Meier survival curves were generated to evaluate the impact of the HALP score on overall survival. Patients were categorized into low and high HALP groups. A statistically significant difference in survival was observed between the groups (*P* = .003), with patients in the high HALP group demonstrating superior overall survival compared to those in the low HALP group. These findings suggest that the HALP score may serve as a prognostic indicator in this cohort. HALP = hemoglobin, albumin, lymphocyte, and platelet.

As shown in Table 2, ROC curve analysis to determine the predictive power of the HALP score for discriminating mortality revealed an area under the curve (AUC) of 0.581 (95% confidence interval [CI]: 0.528–0.633; *P* = .003). The optimal cutoff value for HALP was ≤27.68, with a sensitivity of 57.6% and specificity of 57.5%. The HALP score was statistically significant in discriminating mortality (*P* = .003). The median OS and PFS were calculated; 5‑year OS and PFS rates were 49.4% and 19.2%, respectively (Tables 3 and 4). Kaplan–Meier analysis (Table 3) and (Fig. 1) demonstrated an overall median OS of 60.0 months (95% CI: 51.26–68.74). Significant differences in OS were observed according to the ASCT status (*P* < .001) and HALP score (*P* < .001). As shown in Table 4, the overall median PFS was 23.00 months (95% CI: 18.79–27.20). Age (*P* = .037), sex (*P* = .018), renal dysfunction (*P* = .001), and ASCT status (*P* < .001) were significantly associated with PFS. As shown in Table 5, ASCT and HALP scores were significant variables in the univariate analyses (Table 3). Variables that were significant in univariate analyses were subsequently included in the multivariate Cox regression model. According to the model results, an HALP score ≤27.68 increased the mortality risk by 1.74-fold (hazard ratio [HR]: 1.74, 95% CI: 1.32–2.29, *P* < .001). However, undergoing ASCT once (HR: 0.66, 95% CI: 0.50–0.87, *P* = .004) or twice (HR: 0.28, 95% CI: 0.11–0.71, *P* = .007) reduced the mortality risk. Age, sex, renal dysfunction, and ASCT were significant variables in the univariate analyses (Table 4). Variables that were significant in univariate analyses were subsequently included in the multivariate Cox regression model. It was found that female sex (HR: 0.75, 95% CI: 0.59–0.94, *P* = .013) and, undergoing ASCT once (HR: 0.49, 95% CI: 0.38–0.62, *P* < .001), and twice (HR: 0.47, 95% CI: 0.26–0.85, *P* = .012) reduced the risk of progression (Table 6).

**Table 2:** Predictive value of the HALP score in discriminating mortality.

Variables	AUC	95% CI	Cutoff	Sensitivity (%)	Specificity (%)	*P*
HALP	0.581	0.528–0.633	≤27.68	57.6	57.5	**0.003**

AUC** = **area under the curve, CI = confidence interval, HALP = hemoglobin, albumin, lymphocyte, and platelet.

**Table 3:** Overall survival analysis.

Variables	2-yr %	5-yr %	Median (95% CI)	*P*
Overall	76.6	49.4	60.0 (51.26–68.74)	
Age				
≤65	79.7	54.6	70.0 (56.12–83.87)	.084
>65	73.3	43.1	52.0 (42.37–61.62)	
Sex				
Male	77.7	49.7	60.0 (42.81–77.18)	.401
Female	75.4	49.0	60.0 (50.17–69.82)	
Renal dysfunction				
No	79.3	48.7	59.0 (45.94–72.05)	.276
Yes	73.5	50.6	61.0 (48.72–73.37)	
R-ISS stage				
Stage I	72.4	38.5	51.0 (32.08–69.92)	.447
Stage II	83.3	56.2	73.0 (45.46–100.53)	
Stage III	74.8	49.4	60.0 (49.29–70.70)	
ASCT				
Not performed	66.9	35.7	46.0 (36.58–55.41)	<.001
Performed once	82.7	54.8	73.0 (59.07–86.92)	
Performed twice	100.0	87.5	128.0 (102.58–153.41)	
HALP score				
>27.68	84.4	60.6	79.0 (54.52–103.47)	<.001
≤27.68	68.7	37.6	50.0 (41.36–58.63)	

Kaplan–Meier log rank test, *P* < .05 statistically significant.

CI = confidence interval, HALP = hemoglobin, albumin, lymphocyte, and platelet, R-ISS = revised international staging system.

**Table 4:** Progression-free survival analysis.

Variables	2-yr %	5-yr %	Median (95% CI)	*P*
Overall	49.3	19.2	23.0 (18.79–27.20)	
Age				
≤65	53.6	22.0	25.0 (20.88–29.11)	.037
>65	43.8	15.3	17.0 (11.47–22.52)	
Sex				
Male	41.1	16.9	17.0 (13.22–20.77)	.018
Female	57.5	19.5	29.0 (24.65–33.34)	
Renal dysfunction				
No	54.0	21.9	29.0 (22.15–35.84)	.001
Yes	40.6	13.5	19.0 (14.38–23.61)	
R-ISS stage				
Stage I	51.2	23.2	25.0 (17.55–32.44)	.209
Stage II	52.9	16.8	28.0 (19.44–36.55)	
Stage III	46.2	19.1	21.0 (16.19–25.80)	
ASCT				
Not performed	34.2	9.6	10.0 (6.73–13.26)	<.001
Performed once	58.1	24.1	32.0 (27.53–36.64)	
Performed twice	69.2	23.1	35.0 (22.08–47.91)	
HALP score				
>27.68	50.6	17.2	25.6 (20.36–29.63)	.697
≤27.68	46.2	19.8	20.0 (14.75–25.24)	

Kaplan–Meier log rank test, *P* < .05 statistically significant.

ASCT** = **autologous stem cell transplantation, CI = confidence interval, HALP =** **hemoglobin, albumin, lymphocyte, and platelet, R-ISS** = **revised international staging system.

**Table 5:** Multivariate Cox regression results for mortality risk of various clinical variables.

Variables	HR (95% CI)	*P*
ASCT		.001
Not performed	Ref.	
Performed once	0.66 (0.50–0.87)	.004
Performed twice	0.28 (0.11–0.71)	.007
HALP score		
>27.68	Ref.	<.001
≤27.68	1.74 (1.32–2.29)	

2 log likelihood = 2116.78, *P* < .001.

ASCT = autologous stem cell transplantation, CI = confidence interval, HALP = hemoglobin, albumin, lymphocyte, and platelet, HR = hazard ratio.

**Table 6:** Multivariate Cox regression results of progression risk of various clinical variables.

Variables	HR (95% CI)	*P*
Age		
≤65	Ref.	0.363
>65	1.11 (0.87–1.42)	
Sex		
Male	Ref.	.013
Female	0.75 (0.59–0.94)	
Renal dysfunction		
No	Ref.	.159
Yes	1.18 (0.93–1.48)	
ASCT		<.001
Not performed	Ref.	
Performed once	0.49 (0.38–0.62)	<.001
Performed twice	0.47 (0.26–0.85)	.012

−2 log likelihood = 3259.18, *P* < .001.

ASCT** = **Autologous stem cell transplantation CI = confidence interval, HR = hazard ratio.

Among the components of HALP, albumin (*P* < .001) and hemoglobin (*P* = .001) levels were significantly higher in the high HALP group, whereas lymphocyte and platelet counts were not significantly different.

As shown in Table 1, patients with a low HALP score (≤27.68) were significantly older (*P* = .002) and had lower platelet counts (*P* < .001). Conversely, albumin (*P* < .001), hemoglobin (*P* = .001), and follow-up time were significantly greater in the highHALP group (>27.68). Renal dysfunction (*P* = .008), ASCT status (*P* = .001), disease progression (*P* = .022), and mortality (*P* = .001) also differed significantly between the HALP groups.

As shown in Table 2, ROC curve analysis to determine the predictive power of the HALP score for discriminating mortality revealed an AUC of 0.581 (95% CI: 0.528–0.633; *P* = .003). The optimal cutoff value for HALP was ≤27.68, with a sensitivity of 57.6% and specificity of 57.5%. The HALP score was statistically significant in discriminating mortality (*P* = .003). The median OS and PFS were calculated; 5-year OS and PFS rates were 49.4% and 19.2%, respectively (Tables 3 and 4).

Kaplan–Meier analysis (Table 3) demonstrated an overall median OS of 60.0 months (95% CI: 51.26–68.74). Significant differences in OS were observed according to the ASCT status (*P* < .001) and HALP score (*P* < .001).

As shown in Table 4, the overall median PFS was 23.00 months (95% CI: 18.79–27.20). Age (*P* = .037), sex (*P* = .018), renal dysfunction (*P* = .001), and ASCT status (*P* < .001) were significantly associated with PFS.

As shown in Table 5, ASCT and HALP scores were significant variables in the univariate analyses (Table 3). Variables that were significant in univariate analyses were subsequently included in the multivariate Cox regression model. According to the model results, a HALP score ≤ 27.68 increased the mortality risk by 1.74-fold (HR: 1.74, 95% CI: 1.32–2.29, *P* < .001). However, undergoing ASCT once (HR: 0.66, 95% CI: 0.50–0.87, *P* = .004) or twice (HR: 0.28, 95% CI: 0.11–0.71, *P* = .007) reduced the mortality risk.

As shown in Table 6, age, sex, renal dysfunction, and undergoing ASCT were significant variables in univariate analyses (Table 4). Variables that were significant in univariate analyses were subsequently included in the multivariate Cox regression model. It was found that female gender (HR: 0.75, 95% CI: 0.59–0.94, *P* = .013), undergoing ASCT once (HR: 0.49, 95% CI: 0.38–0.62, *P* < .001), and twice (HR: 0.47, 95% CI: 0.26–0.85, *P* = .012) reduced progression risk. Variables that were statistically significant in the univariate analyses (*P* < .05) were included in the multivariate Cox regression analysis. The R-ISS stage was evaluated in univariate Kaplan–Meier analyses but did not reach statistical significance for OS or PFS; therefore, it was not incorporated into the multivariate model.

## 4. Discussion

The HALP score is an immune-nutritional biomarker calculated using hemoglobin, albumin, lymphocyte, and platelet counts. In recent years, particularly among patients with malignancies, the association between HALP score and prognosis has garnered considerable interest. One of the HALP components, anemia, arises in cancer patients due to factors such as inflammation mediated by pro-inflammatory cytokines, such as interleukin-6, suppression of erythropoiesis, or iron deficiency secondary to chronic blood loss. However, in MM, the pathogenesis of anemia is more complex and differs from that observed in solid tumors. In addition to inflammation-related mechanisms, anemia in MM is largely driven by bone marrow infiltration by malignant plasma cells and alterations in the bone marrow microenvironment, leading to impaired erythropoiesis. In cancer patients, serum albumin reflects nutritional and metabolic status and has been demonstrated to correlate with prognosis. Lymphocytes play a crucial immunological role in tumor surveillance and elimination, and the prognostic significance of lymphopenia in malignancy is well established. Platelets influence tumor angiogenesis by releasing vascular endothelial growth factor and play a role in metastatic spread.^[5,11]^

In MM, the interpretation of the HALP score should be considered within the unique biological and clinical context of this hematologic malignancy. Unlike solid tumors, where HALP primarily reflects systemic inflammatory and nutritional status, MM is characterized by bone marrow infiltration, immune dysregulation, and frequently coexisting anemia and hypoalbuminemia, which are directly related to tumor burden and marrow dysfunction. In this setting, the prognostic contribution of individual HALP components appears to be heterogeneous. Hemoglobin and albumin are well-established prognostic markers in MM, as reflected in their incorporation into the Durie–Salmon staging system and the ISS/R-ISS, and largely reflect disease burden and systemic involvement. In contrast, lymphocyte counts, although associated with immune competence, have shown a less consistent prognostic impact, and platelet counts are often influenced by bone marrow infiltration, tending to decrease in more advanced disease stages. Notably, a lower platelet count, which mathematically increases the HALP score, may paradoxically be associated with more aggressive disease biology in MM. Consistent with this, our results demonstrated that platelet and lymphocyte counts contributed less to the overall prognostic performance of the HALP score compared to hemoglobin and albumin. Therefore, the prognostic value of HALP in MM may be primarily driven by its hemoglobin and albumin components, while the contribution of lymphocyte and platelet counts appears to be more limited and context-dependent. Collectively, these findings suggest that HALP in MM functions as a composite biomarker integrating tumor burden, nutritional status, and host immunity, but with unequal weighting of its components in this disease-specific context.^[2,5]^

In line with these considerations, in our cohort, albumin and hemoglobin levels were the primary contributors to the difference observed between high and low HALP groups, whereas lymphocyte and platelet counts did not differ significantly. This finding may suggest that, in MM, the prognostic impact of HALP is driven predominantly by its nutritional and anemia-related components. However, the composite nature of the HALP score may still provide a more comprehensive reflection of the immuno-nutritional status than any single parameter alone, as it integrates multiple biological domains. Given that hemoglobin and albumin appear to be the main drivers of the HALP score in our cohort, the potential prognostic value of a simplified score incorporating only these two parameters warrants further investigation. Future studies comparing such simplified models with the full HALP score may help clarify whether similar prognostic performance can be achieved with fewer variables.

Because cancer patients have increased metabolic demands and, in addition to calorie deficits caused by anorexia from systemic oncologic treatments (e.g., chemotherapy), are at risk for a chronic catabolic state/cachexia, immune-nutritional status is a critical concern. The HALP score has been investigated in various solid tumors, including gastric, colorectal, bladder, prostate, renal, esophageal, pharyngeal, lung, breast, and cervical cancers.^[5]^ Chen et al assessed the prognostic impact of preoperative HALP in 1332 gastric cancer patients and established a cutoff value of 56.8. The 3-year OS rate was 59.7% in patients with low HALP scores and 74.7% in those with high HALP scores. Patients with high HALP scores not only exhibit smaller tumor sizes but also have less advanced disease.^[13]^

Previous studies have reported conflicting data regarding the relationship between HALP scores and age. Eight studies identified significant differences in HALP by age; 5 reported decreasing HALP with advancing age, while 2 observed the inverse. Fifteen studies observed no significant association with age. It has been suggested that HALP may be prognostically relevant in elderly cohorts but loses significance when all age groups are evaluated together.^[5,14]^ In the present study, although age was associated with survival, no significant relationship was observed between the HALP score and age.

The HALP score, which integrates hemoglobin, albumin, lymphocyte, and platelet levels, has emerged as a promising immuno-nutritional marker with potential prognostic significance in various malignancies. Recent studies have highlighted its relevance in diverse cancer types and medical fields. For example, Dong et al^[15]^ reported its utility in the NHANES cohort, while Ata et al^[16]^ demonstrated its prognostic value in metastatic melanoma patients receiving immunotherapy. Similarly, Zhao et al^[17]^ and Demir et al^[18]^ documented its significance in early breast and pancreatic cancers, respectively, and Büyük et al^[19]^ documented its significance in laryngeal cancer. A systematic review by Xu et al^[6]^ confirmed the emerging role of HALP as a prognostic and immuno-nutritional marker. Beyond oncology, applications have been reported in surgical and neurosurgical settings, as in Tarle et al^[20]^ and Raguž et al,^[21]^ demonstrating the broader applicability of HALP across medical disciplines. These findings support the investigation of the prognostic value of HALP in MM, although its utility remains to be fully elucidated.

Despite the growing interest in the HALP score in the scientific literature, its prognostic impact in hematologic malignancies remains unclear. Vlatka et al retrospectively evaluated the prognostic effect of the HALP score in 153 patients diagnosed with diffuse large B-cell lymphoma (DLBCL), establishing a cutoff value of 20.8. They found that a low HALP score was associated with adverse clinical features in DLBCL and demonstrated that HALP was an independent prognostic index, regardless of stage and international prognostic index.^[9]^ Martinez et al evaluated 1407 Latin American DLBCL patients and determined a HALP cutoff value of ≤13. They identified associations between low HALP scores and female sex, Eastern Cooperative Oncology Group performance status >1, >1 extranodal site, elevated LDH, advanced stage, NLR > 4, and hypoalbuminemia.^[22]^ Patir et al examined 147 patients with Hodgkin lymphoma and identified a cutoff value of ≤19.9 for the HALP score. In early-stage favorable patients, a high HALP score correlated significantly with low NLR, high LMR, and low PLR, although HALP was not statistically significant for disease-free survival or OS across the NLR, LMR, and PLR subgroups.^[10]^ Gursoy et al investigated the prognostic effect of HALP in 130 patients with myelodysplastic syndromes (MDS) and identified a cutoff value of >67.5. They observed higher HALP scores in intermediate-risk International Prognostic Scoring System, as well as in high- and very-high-risk Revised International Prognostic Scoring System MDS patients and those receiving azacitidine. OS was significantly reduced in patients with high HALP scores, and the authors suggested that HALP may be useful for predicting OS and mortality in MDS. In contrast to these findings, in our MM cohort, lower HALP scores were associated with poorer survival, which may reflect disease-specific differences in the biological and clinical significance of the HALP components.^[11]^

However, the relationship between MM and HALP scores remains underexplored. In a study by Solmaz et al involving 200 patients with MM, a HALP score cutoff of 28.8 was identified. They assessed the impact of NLR, PLR, HALP score, and ISS stage on OS and found that patients with a high HALP score had significantly longer OS than those with a low score. They proposed that the HALP score could serve as a novel prognostic marker for MM.^[23]^ Zhang et al evaluated 62 patients with MM and determined a HALP cutoff value of 41. Patients were divided into high HALP (HALP > 41, n = 25) and low HALP (HALP ≤ 41, n = 37) groups. The median OS was 29 months in the high HALP cohort and 20 months in the low HALP cohort, with a statistically significant difference between the 2 groups. Furthermore, among patients with R-ISS stage III disease, those with a high HALP score had a significantly longer median OS than those with a low HALP score.^[24]^ In the present study, we evaluated the relationship between the HALP score and survival and prognosis in 450 patients with MM. Our cohort was larger than that of previous single-center studies, which were drawn from 2 university hospitals. We determined the HALP score according to the ROC analysis in the study. The cutoff value of 27.68 for HALP was determined using ROC analysis within our cohort. However, no external or internal cross-validation was performed, which may increase the risk of overfitting. Future studies with independent cohorts or internal validation methods, such as bootstrapping, are needed to confirm the optimal cutoff and ensure the generalizability of our findings. The HALP score cutoff value in our study was lower than that reported by Zhang et al. This may be due to the small number of patients in the study by Zhang et al and the fact that the studies were conducted in different regions. Our study is similar to that of Solmaz et al study in terms of the HALP score cutoff point from Anatolia. This study represents the largest patient cohort to date to evaluate the prognostic utility of the HALP score in MM. We found that the HALP score was a statistically significant predictor of mortality. The 5-year OS rate was significantly higher in patients with a high HALP score than in those with a low score (60.6% vs 37.6%). The median OS was 79 months in the high-HALP group and 50 months in the low-HALP group, confirming the prognostic significance of the HALP score for OS. Although the difference was not statistically significant, the median PFS was 25.6 months in the high-HALP group and 20 months in the low-HALP group. In our study, PFS did not differ significantly between the HALP groups (*P* = .697), and HALP was not identified as an independent predictor of PFS in multivariate analyses. Therefore, while HALP may provide complementary prognostic information for OS, its utility in predicting PFS was limited within this cohort. These findings suggest that HALP should be interpreted with caution when considering disease progression outcomes, and further studies are needed to clarify its role in PFS prognosis. Age, sex, renal dysfunction, and undergoing ASCT were significant variables in univariate analyses. Variables that were significant in univariate analyses were subsequently included in the multivariate Cox regression model. It was found that female sex (*P* = .013) and undergoing ASCT once (*P* < .001) and twice (*P* = .012) reduced progression risk.

Although the HALP score exhibited only modest discriminative ability in the ROC analysis (AUC = 0.581), it was statistically significant. This indicates that HALP alone is not a robust prognostic marker and is most useful as a complementary parameter, particularly when integrated into composite prognostic models rather than when applied independently.

The HALP score remained a statistically significant and independent prognostic factor for OS in the multivariate analysis (HR: 1.74, *P* < .001), underscoring its clinical relevance. The relatively low AUC may reflect the complex biology of MM and the multifactorial nature of survival in these patients. However, HALP’s statistical significance and independent predictive value of HALP highlights its potential as a clinically useful biomarker when integrated into broader risk models. Furthermore, the mortality risk was 1.74 times higher in patients with low HALP scores. Undergoing ASCT once or twice reduced both mortality and the risk of progression. Our results demonstrate that a low HALP score is associated with an increased risk of mortality, whereas ASCT is associated with reduced risks of both mortality and disease progression. This study included the largest patient cohort to date and clearly demonstrated that lower HALP scores were associated with poorer prognosis in MM patients. Although platelet count is inversely incorporated in the HALP formula, the overall HALP score reflects the combined effect of all 4 parameters; in our cohort, the concomitant reductions in hemoglobin, albumin, and lymphocyte levels likely outweighed the mathematical contribution of platelet count, resulting in lower HALP scores in patients with more advanced disease.

Recent large-scale and disease-specific studies published in 2025 further support the growing prognostic relevance of the HALP score across different malignancies. Qin Li et al^[7]^ demonstrated in a comprehensive cohort of patients with non-small cell lung cancer that low HALP scores were significantly associated with adverse clinicopathological features and inferior survival outcomes, emphasizing the role of immune-nutritional imbalance in tumor progression and prognosis. Similarly, Na Li et al^[12]^ reported that the HALP score was an independent prognostic factor for survival even in low-risk and early-stage extranodal natural killer/T cell lymphoma, highlighting its utility beyond advanced disease settings and reinforcing its value in hematologic malignancies. These findings align with our results, suggesting that the HALP score reflects a systemic host–tumor interaction that is relevant across both solid and hematologic cancers. In this context, our study extends the existing literature by providing the largest cohort to date evaluating the prognostic significance of HALP in MM, further supporting its role as a simple, accessible, and clinically meaningful prognostic biomarker. However, differences in optimal cutoff values and disease-specific outcomes across studies underscore the need for standardized validation and prospective investigations.

Our study cohort spans 2008 to 2023, a period during which treatment strategies for MM have substantially evolved, including the introduction of daratumumab, lenalidomide maintenance, CAR-T therapy, and bispecific antibodies. This treatment heterogeneity may have influenced survival outcomes and could partially confound the associations between HALP levels and prognosis. While major therapy types and transplantation status were considered in the baseline analyses, detailed treatment stratification was not feasible due to sample size and retrospective data limitations. Therefore, the prognostic value of HALP should be interpreted within the context of evolving therapeutic approaches, and future studies with more homogeneous treatment cohorts or stratified analyses are required to validate these findings.

This study had several important limitations. Although it included a larger patient cohort than previous studies, it remains a retrospective analysis involving only 2 centers, 1 in Western Anatolia and 1 in Eastern Anatolia. Its retrospective nature and lack of standardized data collection across the 2 participating institutions may have introduced selection and information bias. Patient inclusion criteria may have differed slightly between centers, leading to heterogeneity in baseline characteristics. Similarly, treatment strategies, including induction regimens, supportive care, and access to novel agents, were not fully harmonized across institutions and may have influenced survival outcomes independently of HALP status. In addition, laboratory assessments, such as hemoglobin, albumin, lymphocyte, and platelet counts, were not performed under a centralized protocol, and variations in timing, methodology, or reference standards could have affected the HALP calculation. These factors limit the generalizability of our findings and should be carefully considered when interpreting the results. Future prospective, multicenter studies with broader geographic representation are needed to better determine the generalizability and prognostic relevance of the HALP score.

## 5. Conclusion

Despite the marked improvement in survival with novel therapies, MM remains an incurable disease. Established prognostic factors for MM include disease stage, cytogenetic risk, and patient age. The HALP score, a relatively recently defined immune-nutritional biomarker, has generated interest for its potential prognostic value. The present study demonstrated that a high HALP score correlates with improved OS, whereas a low HALP score is associated with increased mortality risk. Thus, along with existing parameters, the HALP score may serve as a novel prognostic biomarker for MM. We anticipate that future prospective studies will further validate the long-term reliability and validity of the HALP scores.

In conclusion, our findings suggest that the HALP score may serve as a promising complementary prognostic parameter in patients with MM. However, given the modest discriminative ability observed (AUC = 0.581) and the retrospective design of this study, HALP should not be used in isolation for clinical decision-making. Prospective studies and external validation are needed to confirm its prognostic value and determine its potential role in composite risk models.

## Author contributions

**Conceptualization:** Gulsum Akgun Cagliyan, İlhami Berber.

**Data curation:** Gulsum Akgun Cagliyan, Eda Nilufer Coskun, Suleyman Arslan, Huriye Karagul, Seher Yilmaz, Ismail Can Kendir, Mehmet Ali Erkurt, Aybuke Nur Ekici, Mehmet Nuri Baser.

**Formal analysis:** Gulsum Akgun Cagliyan.

**Funding acquisition:** Gulsum Akgun Cagliyan.

**Investigation:** Gulsum Akgun Cagliyan, İlhami Berber, Eda Nilufer Coskun, Suleyman Arslan, Huriye Karagul, Emin Kaya, Ismail Can Kendir, Mehmet Ali Erkurt, Mehmet Nuri Baser, Irfan Kuku.

**Methodology:** Gulsum Akgun Cagliyan, Huriye Karagul, Seher Yilmaz, Emin Kaya, Basak Unver Koluman, Sibel Hacioglu, Mehmet Nuri Baser, Irfan Kuku.

**Project administration:** Gulsum Akgun Cagliyan.

**Resources:** Gulsum Akgun Cagliyan, Basak Unver Koluman.

**Software:** Gulsum Akgun Cagliyan.

**Supervision:** Gulsum Akgun Cagliyan.

**Validation:** Gulsum Akgun Cagliyan.

**Visualization:** Gulsum Akgun Cagliyan.

**Writing – original draft:** Gulsum Akgun Cagliyan.

**Writing – review & editing:** Gulsum Akgun Cagliyan.
